# Suicide patterns on the London Underground railway system, 2000–2010

**DOI:** 10.1192/pb.bp.115.052050

**Published:** 2017-10

**Authors:** Susanna Martin, Muffazal Rawala

**Affiliations:** 1 Oxleas NHS Foundation Trust; 2 Luton Mental Health and Wellbeing Service, East London NHS Foundation Trust

## Abstract

**Aims and Method** Suicidal acts on underground railway networks are an area of public health concern. Our aim was to review recent epidemiological patterns of suicidal acts on the London Underground to inform future preventive interventions. Data from 2000 to 2010 were obtained from the British Transport Police via a Freedom of Information request.

**Results** The mean annual rate of suicidal acts from 2000 to 2010 was 5.8 per 100 million passenger journey stages. Of those who died by suicide, 77.3% were of White Northern European ethnicity. A fifth had a history of mental illness.

**Clinical implications** The widening gap between the number of recorded suicide attempts and completed suicides is encouraging. Further research is required regarding the role of drug and alcohol use, psychiatric history and area of residence. Installation of platform screen doors should be considered in future railway network expansion.

The London Underground was built in 1863 End was the world's first underground railway system.^1^ Today it comprises 11 train lines serving a total of 270 stations across 402 km.^1^ In 2015/16 it saw the completion of 1.34 billion journeys across its network.^1^ Suicide on underground railway systems is a major cause for public health concern. Railway suicide can have a traumatic impact on both train drivers^2^ and witnesses, and can lead to significant train delays, with substantial economic consequences.^3^

Between 1940–49 and 1980–89 the mean number of suicidal acts per year on the London Underground rose from 36.1 to 94.1.^4^ Previous studies have suggested an association between the rising number of suicidal acts across the national railway and increasing volumes of passengers across railway networks;^5,6^ however, this finding has been inconsistent across studies.^7^ Use of preventive measures to stop suicidal acts on railways has been the focus of several studies, for example, concentrating on changes to the immediate environment, media reporting and interagency collaborations.^8^

Our aim was to review recent epidemiological patterns of suicidal acts on the London Underground to better inform future preventive interventions.

## Method

Data covering deaths by suicide between 2004 and 2010 and ‘person under train’ incidents between 2000 and 2010 were obtained by one of the authors (M.R.) on 7 October 2011 through a Freedom of Information request to the British Transport Police. Data covering completed suicides between 2000 and 2003 could not be provided owing to changes in coding. No further information in relation to this was provided by the British Transport Police.

The data provided by the British Transport Police included demographic information (age, gender, ethnicity) of suicide victims between 2004 and 2010. Age was categorised as: <15 years, 15–44 years, 45–74 years and > 75 years. The authors also received figures pertaining to all suicide attempts (which included incidents where individuals were prevented from jumping in front of a train or accessing the tracks) and all ‘person under train’ incidents (intentional and accidental) between 2000 and 2010.

Information regarding the total number of journeys completed on the London Underground was obtained from the publicly accessible Transport for London (TfL) 2011 *Travel in London* report.^9^ Passenger journeys were recorded as ‘journey stages’, where a journey stage represents a segment of a trip made on a particular mode of transport. For example, a journey made up of two stages could include a walking stage to the Underground station followed by a second stage on the Underground network.^10^

## Results

Between 2000 and 2010 there were 644 recorded suicide attempts on the London Underground. The mean annual rate of suicide attempts during this period was 5.8 per 100 million journey stages (95% CI 5.0–6.5). Between 2004 and 2010 there were 132 deaths by suicide. The mean annual rate of individuals who died by suicide during this period was 1.8 per 100 million journey stages (95% CI 1.4–2.2). In addition, there were 38 deaths in which the coroner recorded an open verdict and 9 deaths in which the inquest had not yet taken place or in which the British Transport Police did not have access to the outcome of the verdict. The total number of ‘person under train’ incidents between 2000 and 2010, which included both intentional and accidental acts, was 433. The mean annual rate of ‘person under train’ incidents during this period was 3.9 per 100 million journey stages (95% CI 3.6–4.2).

**Fig. 1:**
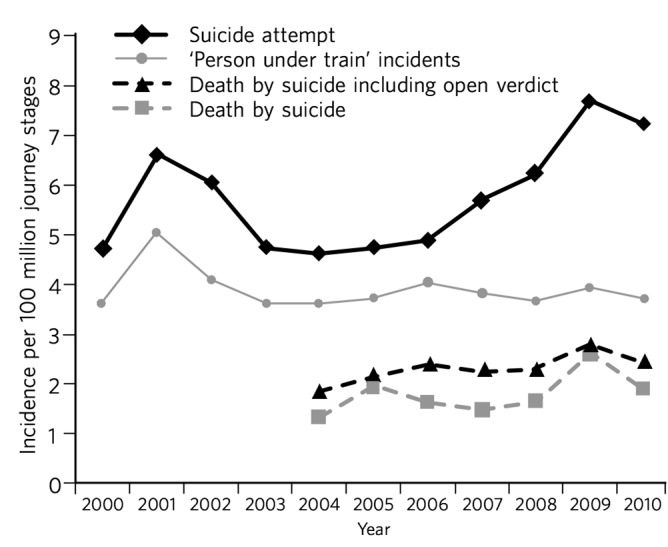
Suicide attempts *v*. deaths by suicide, 2000–2010.

**Fig. 2:**
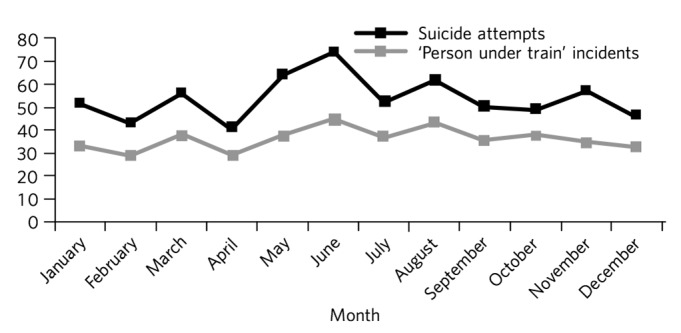
Suicide attempts and ‘person under train’ incidents by month, 2000–2010.

Figure 1 shows an increase in the number of suicide attempts from 2004 to 2009, which is more marked than the increase in the number of incidents of death by suicide. The number of ‘person under train’ incidents, however, remained relatively constant. Our data revealed a small increase in the number of suicide attempts and ‘person under train’ incidents during May–August, with a peak in June, as illustrated in Fig. 2.

Tables 1 and 2 display the age and ethnicity distribution for males and females in incidents of deaths by suicide 2004–2010. Age at death was not available for two male individuals, and ethnicity data were not available for one male individual. Of the deaths by suicide, two-thirds (*n* = 88) were male and a third (*n* = 44) were female. The average age at death was 40.7 years in males and 45.5 years in females.

**Table 1:** Age and ethnicity in incidents of death by suicide in males, 2004–2010^a^

	Age, years: *n* (%)					Ethnicity, *n* (%)					
	<15	15–44	45–74	>75	Total	White North European	White South European	Black	Asian	Middle Eastern	Total
2004	0 (0.0)	8 (66.7)	4 (33.3)	0 (0.0)	12	6 (50.0)	0 (0.0)	3 (25.0)	3 (25.0)	0 (0.0)	12

2005	0 (0.0)	4 (66.7)	1 (16.7)	1 (16.7)	6	5 (71.4)	0 (0.0)	0 (0.0)	2 (28.6)	0 (0.0)	7

2006	0 (0.0)	9 (69.2)	4 (30.8)	0 (0.0)	13	11 (84.6)	0 (0.0)	1 (7.7)	0 (0.0)	1 (7.7)	13

2007	0 (0.0)	10 (76.9)	2 (15.4)	1 (7.7)	13	12 (92.3)	0 (0.0)	1 (7.7)	0 (0.0)	0 (0.0)	13

2008	0 (0.0)	10 (76.9)	3 (23.1)	0 (0.0)	13	9 (69.2)	0 (0.0)	1 (7.7)	3 (23.1)	0 (0.0)	13

2009	0 (0.0)	10 (62.5)	5 (31.3)	1 (6.3)	16	12 (75.0)	0 (0.0)	3 (18.8)	1 (6.3)	0 (0.0)	16

2010	0 (0.0)	6 (46.2)	7 (53.8)	0 (0.0)	13	10 (76.9)	0 (0.0)	0 (0.0)	3 (23.1)	0 (0.0)	13

Total	0 (0.0)	57 (66.3)	26 (30.2)	3 (3.5)	86	65 (74.7)	0 (0.0)	9 (10.3)	12 (13.8)	1 (1.1)	87

a. Age not available for one individual; ethnicity data not available for two individuals.

**Table 2:** Age and ethnicity in incidents of death by suicide in females, 2004–2010

	Age, years: *n* (%)					Ethnicity, *n* (%)					
	<15	15–44	45–74	>75	Total	White North European	White South European	Black	Asian	Middle Eastern	Total
2004	0 (0.0)	0 (0.0)	0 (0.0)	1 (100.0)	1	1 (100.0)	0 (0.0)	0 (0.0)	0 (0.0)	0 (0.0)	1

2005	0 (0.0)	6 (54.5)	5 (45.5)	0 (0.0)	11	10 (90.9)	0 (0.0)	0 (0.0)	1 (0.1)	0 (0.0)	11

2006	0 (0.0)	1 (25.0)	2 (50.0)	1 (25.0)	4	4 (100.0)	0 (0.0)	0 (0.0)	0 (0.0)	0 (0.0)	4

2007	0 (0.0)	1 (33.3)	2 (66.7)	0 (0.0)	3	3 (100.0)	0 (0.0)	0 (0.0)	0 (0.0)	0 (0.0)	3

2008	0 (0.0)	4 (80.0)	1 (20.0)	0 (0.0)	5	3 (60.0)	0 (0.0)	1 (20.0)	1 (20.0)	0 (0.0)	5

2009	0 (0.0)	5 (41.7)	7 (58.3)	0 (0.0)	12	9 (75.0)	0 (0.0)	1 (8.3)	2 (16.7)	0 (0.0)	12

2010	0 (0.0)	5 (62.5)	2 (25.0)	1 (12.5)	8	7 (87.5)	1 (12.5)	0 (0.0)	0 (0.0)	0 (0.0)	8

Total	0 (0.0)	20 (50.0)	19 (43.2)	3 (6.8)	44	37 (84.1)	1 (2.3)	2 (4.5)	4 (9.1)	0 (0.0)	44

Of the 132 individuals who completed suicide on the London Underground, 110 (83.3%) were resident in London at the time of the event. Information was unavailable for one individual. A history of mental illness was confirmed in 20.5% of individuals (*n* = 27).

The Northern Line had the greatest number of recorded suicide attempts (*n* = 145) between 2000 and 2010, followed by the Central Line (*n* = 99) and Piccadilly Line (*n* = 92). The lowest numbers of suicide attempts during that period were recorded on the Jubilee Line (*n* = 27) and Bakerloo Line (*n* = 33).

**Fig. 3:**
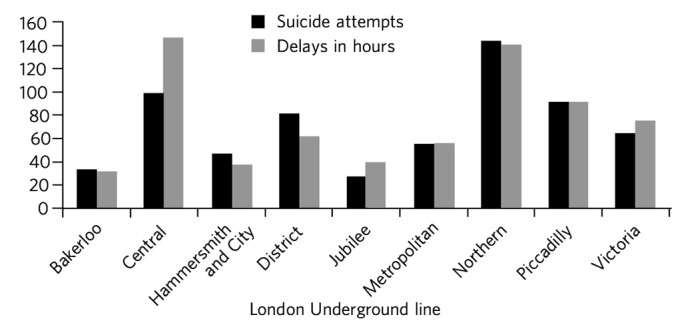
Total number of suicide attempts per line and delays (in hours), 2000–2010,

King's Cross St Pancras station saw the highest number of suicide attempts between 2000 and 2010 (*n* = 18), followed by Mile End (*n* = 17), Victoria (*n* = 16), Camden Town (*n* = 15), Archway (*n* = 13), Liverpool Street (*n* = 13), Oxford Circus (*n* = 12), Green Park (*n* = 12) and Earl's Court (*n* = 11). Suicide attempts led to delays in underground railway services; between 2000 and 2010 the Northern Line and Piccadilly Line experienced the greatest total delays (8484 min and 5521 min, respectively). The Jubilee Line recorded total delays of 2396 min and the Bakerloo Line 1911 min over the same period (Fig. 3).

## Discussion

### Suicide patterns over time

There was a marked increase in the number of suicide attempts across the London Underground from 2004 onwards, although this trend was beginning to reverse in 2009. Although there was also a rise in deaths by suicide during that period, this was of a lesser degree. In their 2016 paper looking at suicide trends across the entire England and Wales railway system, Taylor *et al* found that male suicide rates had increased from 6 to 8.4 per million from 2000 to 2013, with the greatest increase observed between 2010 and 2013, from 6.4 to 8.4 per million.^11^ By contrast, female suicide rates remained relatively constant. Although previous studies looking at suicide attempts on underground railway systems have suggested a case fatality rate of 43 to 55%^4,12,13^ (compared with 90% on overground railways^14^), this does not fully explain the extent of the difference observed. Increased reporting of suicide attempts by London Underground staff may be a factor, as well as the introduction of preventive interventions allowing for the early identification of persons at risk.

TfL has worked towards suicide prevention since the 1990s.^15^ This has included strategically placed Samaritans campaign posters and telephones within stations. More recently there has been further collaborative work with the Samaritans to train staff in identifying persons at risk, and giving them the confidence to intervene.^16^ Additional interventions have included platform-edge barriers on the Jubilee Line and gates to prevent passengers from entering tunnels at existing stations, as well as use of video surveillance and markings/warning signs to prevent individuals from approaching the platform edge.^15^ Presence of staff on platforms during rush-hour periods may also be helpful. The increase in the number of suicide attempts and deaths by suicide in 2008 and 2009 may in part be explained by the financial crisis of 2008.^17^

The number of journey stages made on the London Underground increased from 0.976 million in 2004 to 1.065 million in 2009.^9^ There have been several studies exploring a potential link between higher numbers of passenger journeys and the incidence of suicide, with variable findings. Sonneck *et al* found no such association on the Viennese subway,^7^ whereas in Stockholm, Sweden, and The Netherlands, authors noted a positive association.^5,6^ Although Waterloo station is the busiest in terms of passenger flow,^18^ it does not feature in the top 20 stations for suicide attempts on the London Underground in 2000–2010.

Our data did not reveal any marked variation in suicide attempts across the year, although there was a small increase in events during May to August, with a peak in June. Findings in other studies have been variable. Although Dinkel *et al* found no association between seasons of the year and suicide events,^19^ others have noted a small increase in suicide events during the summer months^4^ or during the months of April and September.^20^ Even though summer months are associated with higher numbers of tourists in London, it is unclear whether this has had an effect on the number of suicide attempts during those months. Although a majority of those who died by suicide on the London Underground were mostly resident in the city, the time of year of their death was not available, nor was the proportion of London residents represented among those individuals who attempted suicide.

### Demographics

The stark overrepresentation of men in the 15- to 44-year age group (61.4%, *n* = 57) among those who died by suicide was in keeping with national figures (England), where the proportion of males is approximately two-thirds.^21^ The overall gender distribution of suicide victims on the London Underground was very similar to that recorded nationally during the same period (2004–2010), with 66.9% of cases being male and 33.1% female. By contrast, the proportion of females aged over 75 years was almost double that of males (6.8% and 3.5% respectively), although the numbers were very small and therefore need to be interpreted with caution.

This is the first study looking at the ethnic distribution of suicide victims on the London Underground. The predominance of individuals of White Northern European background (>75%) is significant in a place like London where people of White ethnic background make up less than 60% of the total population,^22^ in particular since 83.3% of individuals who died by suicide on the London Underground were resident in the city. Moreover, use of the London Underground is evenly distributed across different ethnicities.^23^ There are currently no data available on the ethnicity of individuals who die by suicide nationally, hence we do not know whether individuals of White Northern European ethnicity are also overrepresented relative to other ethnic groups. Similarly, we do not know whether individuals of White Northern European ethnicity are overrepresented in suicide incidents occurring across the rest of the national railway network or involving violent suicide methods.

In our sample a fifth of individuals had a history of mental illness. Unfortunately, no further data were available regarding their diagnosis, level of psychiatric care at the time of suicide or whether they were current or recent in-patients of a psychiatric unit. Proximity of stations or railway suicide ‘hot spots’ to psychiatric hospitals has been highlighted as a risk factor for suicide in several studies,^4,5,12,24,25^ although the result was not found to be significant across the Stockholm railway system.^5^ In their 1987 study of 100 individuals who had attempted suicide on the London Underground, Cocks found that 13 were current psychiatric in-patients, and 2 had been discharged within 48 h of the event.^26^ Fifteen individuals had expressed suicidal ideation in the 24 h preceding the event. Although proximity to a psychiatric hospital was not taken into account in this paper, the presence of a history of mental illness in a fifth of those who died by suicide highlights the need for staff working in psychiatric hospitals to be aware of the proximity of any nearby Underground stations in their assessment of risk in patients.

### Additional risk factors

In Stockholm, authors found that the stations with the highest suicide rates were associated with higher levels of surrounding drug-related crime.^27^ Previous studies have shown that consumption of alcohol or drugs was a characteristic of about 10 to 20% of individuals who had attempted or died by suicide on an underground railway system.^12,28,29^ In the UK National Confidential Inquiry into Suicide and Homicide by People with Mental Illness, the authors noted that 54% of those who died by suicide and had been in contact with mental health services over the past year had a history of alcohol and/or drug misuse.^30^ Intoxication with drugs or alcohol can increase the risk of impulsive and risk-taking behaviour and may have been a contributing factor in the incidents observed on the London Underground, including ‘person under train’ incidents of accidental cause. Unfortunately, the British Transport Police do not currently collect this information. Consumption of alcohol on the London Underground is forbidden; however, consideration must be given as to how to manage the risk of suicidal behaviour or accidental injury in intoxicated individuals.

### Use of preventive measures

Several recent studies looking at suicide attempts on underground and overground railway systems have focused on the use of preventive measures. Diminishing ease of access to the track through barrier methods is suggested as an effective intervention, although Cox *et al* noted a lack of studies demonstrating this.^31^ In Seoul, the presence of platform screen doors (PSDs) reduced suicides on the underground network by 89%.^32^ PSDs are continuous panels that separate the platform from the train, only opening when the train is in the station. Half-length PSDs (1.6 m) were less effective than full-length PSDs; however, full-length PSDs were 120–150% more expensive to install.^32^ The effectiveness of PSDs was also noted in Hong Kong.^33^ Portions of the Jubilee Line include the presence of PSDs, which may be a contributory factor to the lower number of suicide attempts observed on this line relative to the rest of the network. The presence of PSDs would also reduce the risk of accidental falls on to the railway tracks, thus reducing the number of ‘person under train’ incidents. Although the cost of installing these doors makes their presence across the railway network unlikely in the near future, their potential for significantly reducing the number of injuries or deaths on the tracks should be considered when planning future network renovation or expansion.

Other proposed interventions include increasing the presence of drainage pits, as mortality at London Underground stations that have a drainage pit was 44% *v*. 76% at those stations without, in incidents of individuals falling or jumping in front of trains.^34^ These drainage pits were originally constructed to drain water between the tracks in deep-tunnel stations. Installation of blue lights at platforms was put forward as an effective measure by Matsubayashi *et al* in 2013^35^ but this benefit was minimised by Ichikawa *et al* the following year.^36^ Another suggested measure is reducing the average speed of trains entering the station at peak times.^29^ In Vienna, changing the way in which media reported suicides on the underground by making it less dramatic was followed by a reduction in suicide.^7^

### Strengths and limitations

This is the most recent study looking at patterns of suicide on the London Underground since 1994. It is also the first study looking at ethnicity within incidents of death by suicide on the Underground, and the findings raise new and important dimensions when seeking to introduce preventive measures.

There were several limitations to the study: no data were available for deaths by suicide between 2000 and 2003 owing to changes in coding, and death by suicide could not be confirmed in 47 cases owing to the recording of an open verdict or the outcome of the inquest not being available. Because of the relatively rare occurrence of deaths by suicide on the London Underground the numbers used in the study were small, leading to possible sample size bias. The limited breadth of information collected by the British Transport Police meant that certain risk factors (e.g. use of drugs/alcohol or whether individuals were psychiatric in-patients at the time of the event) could not be adequately explored in our sample. The study is further limited by the absence of data beyond 2010.

### Conclusions

Suicide on the London Underground railway system continues to be an important public health issue. The widening gap between the number of recorded suicide attempts and deaths by suicide is encouraging and may reflect the introduction of preventive measures. Use of PSDs has been proven to be effective for both suicidal acts and accidental ‘person under train’ incidents and needs to be considered when planning railway renovation or construction projects. The overrepresentation of people of White Northern European ethnicity among those who attempted suicide requires further exploration, as does the proportion of non-residents involved in suicide attempts on the London Underground across the year to better target preventive interventions. Finally, the collection of data on alcohol and drug use, as well as a more detailed psychiatric history, would help to inform further research and implementation of preventive measures for suicidal acts on the London Underground.
